# Low-Cost Benzene Toluene Xylene Measurement Gas System Based on the Mini Chromatographic Cartridge

**DOI:** 10.3390/s21010125

**Published:** 2020-12-28

**Authors:** Emiliano Zampetti, Paolo Papa, Joshua Avossa, Andrea Bearzotti, Catia Balducci, Giovanna Tranfo, Antonella Macagnano

**Affiliations:** 1 Institute of Atmospheric Pollution Research–National Research Council (IIA-CNR), Research Area of Rome 1, Via Salaria km 29.300, 00015 Monterotondo, Italy; p.papa@iia.cnr.it (P.P.); joshua.avossa@empa.ch (J.A.); a.bearzotti@iia.cnr.it (A.B.); balducci@iia.cnr.it (C.B.); antonella.macagnano@cnr.it (A.M.); 2 Department of Occupational and Environmental Medicine, Epidemiology and Hygiene, National Institute for Insurance against Accidents at Work (INAIL), via Fontana Candida 1, Monte Porzio Catone, 00078 Rome, Italy; g.tranfo@inail.it

**Keywords:** BTX, chromatography, mini chromatography column, low-cost gas measurement system, Arduino

## Abstract

Benzene, toluene and xylene (BTX) are an important part of the volatile organic compounds (VOCs) to be detected and monitored in the air, due to their toxicity towards human health. One of the most reliable technique used in BTX detection is gas chromatography (GC), which presents a high sensitivity. On the other hand, it has important drawbacks, such as high costs, the need for qualified personnel and frequent maintenance. To overcome these drawbacks, this work reports the development of a low cost and portable BTX gas detection system based on a mini chromatographic cartridge, a photo ionization detector (PID), a simple control unit (based on Arduino architecture) and a mini pump. In order to separate the BTX components, we propose the use of a cartridge 80 mm in length, composed of several commercial chromatographic column sections. To test the system performances, we have injected different amounts (from about 0.3 to 5.3 µg) of benzene, toluene and xylene and two of the most frequent possible interferents (ethanol, acetone). Experimental results have shown different retention time values (i.e., 25 ± 0.5 s, 51 ± 1.2 s and 117 ± 4 s, respectively) for benzene, toluene and xylene.

## 1. Introduction

Outdoor and indoor environmental monitoring has had a significant increase in interest due key concern human health. For instance, it is recognized that there is a wide range of compounds in indoor and outdoor air that are of interest because of the possible effects on the health of occupants. Depending on the kind of pollutant and on the concentration and the duration of exposure, some organs can be more affected than others [1]. The most frequent disorders are those caused by irritant gases and particulates on the mucous membranes and respiratory organs [2]. Among the large group of pollutants, VOCs as BTX [3,4] are extremely harmful and dangerous due their toxicity, carcinogenicity and combustibility [5,6,7]. These compounds are produced by human activities, but they are also employed in many industrial fields, among which the following stand out: oil refining, cosmetics and detergents, paints and resins and shipbuilding yards [8,9]. For these reasons, BTX are subject to many directives in several countries regarding the limits of exposure for the personnel involved. There are several standardized and well-known methods to monitor these compounds, including passive samplers and automatic chromatography [10,11,12]. Despite this, in the last years innovative gas sensing system or sensor development remains an active area of research, in order to develop technologies capable to detect gasses increasing the sensitivity, specificity and efficiency and decreasing the cost and dimensions [13,14]. Many studies have been focused on chemo-resistors based on conductive or semi-conductive polymers and metal oxides, using the nanotechnology to obtain higher sensitivity, a lower detection threshold and better selectivity [15,16,17,18,19,20,21]. Other studies investigated nanomaterial or smart material performances deposited on different piezoelectric-based chemo-sensors [22,23,24,25]. Optical VOC systems or sensors have also been extensively studied, but in many cases, they were expensive and suffered from a lack of portability [26,27,28,29,30].

Even if considerable progresses have been obtained regarding the sensitivity, response time and detection limits, the challenge of discriminating the different compounds of a BTX mixture remains open. This feature, together with low cost and portability, are very important in the practice in which the sensor system will work. Currently, among the most-used analytical instrumentations for the measurement and analysis of BTX mixtures, there are chromatographs in both laboratory and portable versions [31,32,33]. Nevertheless, some drawbacks, such as dimensions, time spent for each measurement, need of peculiar carrier gases (e.g., argon, helium) and great maintenance costs, limit the practical applications. Chromatography is an analytical technique based on the principle of the separation of two or several components due to their different distribution between two phases: stationary and mobile (i.e., the carrier). Generally, it is defined as liquid chromatography (LC) when the mobile phase is liquid and GC when the mobile phase is gaseous [34,35,36,37]. Its extreme versatility makes this technique widely used in many fields of analytical chemistry application including: drug, pharmaceutical, food, medical, biological and environmental monitoring [38,39,40,41,42].

In recent years, many research groups and companies have focused on the study and the development of portable systems for measuring BTX, combining new technological products in the field of sensors and micro-mechanics with the chromatography technique. Different technological or method strategies have been used to develop this type of systems, such as commercial columns connected to integrated devices and sensors [43] or micro fabricated columns using dry and wet micromechanics technologies [44,45,46,47]. Although these devices have shown excellent results, their manufacture is complex and does not have a very low cost for a small volumes of production.

A further strategy, the 3D printing technology application, is a more innovative, easy to use and low-cost solution for the production of chromatography integrated columns. Its layer-by-layer production process enables the creation of complex network of channels, voids and overhangs of chromatographic stationary phases, which were previously very difficult to produce [48]. However, there are still many issues related to their reproducibility and to the selection of available materials compatible with chemicals that can be used for actual printing technologies (e.g., fused deposition modeling—FDM, stereo-lithography—SLA, selective laser melting or sintering—SLM). To overcome these issues, some research groups and companies have developed hybrid systems based on commercial columns and detectors, opportunely modified to reduce the overall dimensions [49,50,51,52].

In this work, we present the development and testing of a simple, effective and low cost BTX measurement system based on a chromatographic cartridge using purified environmental air as carrier. The developed cartridge consisted in a mini glass tube (80 mm long and 7 mm in diameter) filled with a proper combination of several segments of a commercial gas chromatographic column. A commercial PID was used to measure the VOC concentrations and a mini membrane pump to implement the sampling system. A simple electronic board (based on low-cost microprocessor like Arduino Nano, www.arduino.cc) acquires the PID signal, controls the cartridge temperature and sends to a personal computer the measurement data. In the experimental section, we report the scheme of the measurement system and the fabrication of the mini cartridge. Finally, the results of experimental measurements with BTX and two common industrial interferents compounds (e.g., acetone, ethanol) are reported.

The novelty of our work is mainly related to the original and simple strategy adopted for the manufacture of a small-size chromatographic cartridge (of a few cubic centimeters), with a very fast separation performance (in the order of seconds), that does not need a vacuum system to operate the compounds separation. In particular, using a parallel disposition of chromatographic columns sections, the resulting cartridge acts as a fast compound separator, and at the same time, its low pneumatic impedance allows to be used with low-cost mini dc pumps, which usually have low pneumatic prevalence. In this letter, we show the first results that encourage the future use of this strategy for the development of mini separation systems based on standard columns available on the market. In the most scientific manuscripts that deal with miniaturization systems for the measurement of BTX, the separation systems are generally built ad hoc by using high-cost technologies.

## 2. Materials and Methods

### 2.1. Measurement System Development

**Figure 1:**
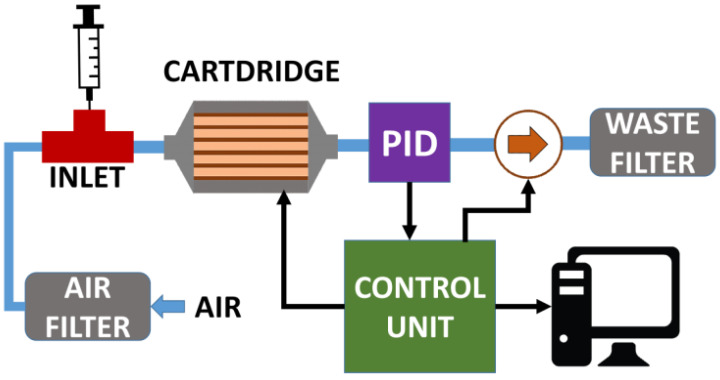
Scheme of the developed system.

A scheme of the newly developed system is presented in Figure 1. The core of the system is the cartridge (Packed Columns Cartridge—PCC) that performs the separation of the three BTX components. The sample, injected into the carrier gas (air) by means of a “T-shape’’ Teflon tubing adapter (INLET), is delivered to a PID (MiniPID 2 HS with a lamp of 10.6 eV by Ion Science) measurement chamber by using a dc mini pump (NMP03KPDC-M by KNF). The air used as carrier gas was generated by filtering the ambient air by a packed active carbon pellets cartridge (AIR FILTER). A microcontroller electronic board (CONTROL UNIT), interfaced with LabVIEW software, managed all the system tasks: (a) the conversion of the analogue PID signal into digital data (with 0.25 s of sample rate); (b) the control of PCC temperature, which can be set at a value ranging from T_ENV_ (environmental temperature) up to 250 °C; (c) the sending of all the data to the personal computer unit, where a LabView software records the data and performs their elaboration.

**Figure 2:**
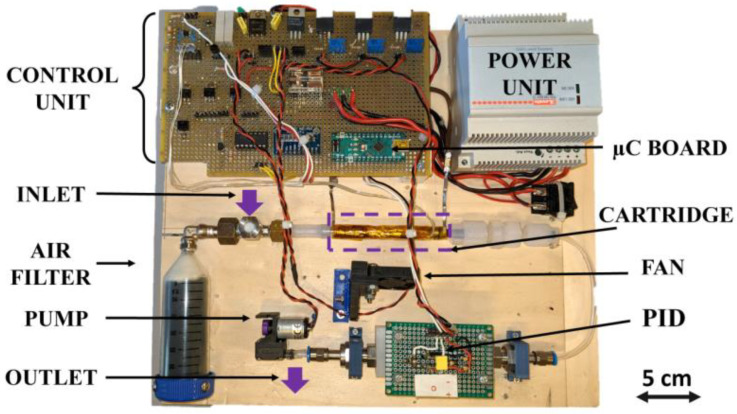
First system prototype.

In particular, the control unit consisted in a microcontroller (µC Board was an Arduino Nano, by Arduino Inc.) connected to an external 16 bit analog to digital converter (ADS1115 from Texas Instruments) through an Inter Integrated Circuit protocol (I^2^C). A suitable designed analog board supplied the power to the electronic circuits and the PID. In this way, the external analog to digital converter ensured that the read-out voltage resolution matched with our needs. In Figure 2, a picture is reported of the first prototype that was developed. After the first tests, some electrical components were changed to optimize the dimensions, the analogue to digital conversion and the power consumption.

**Figure 3:**
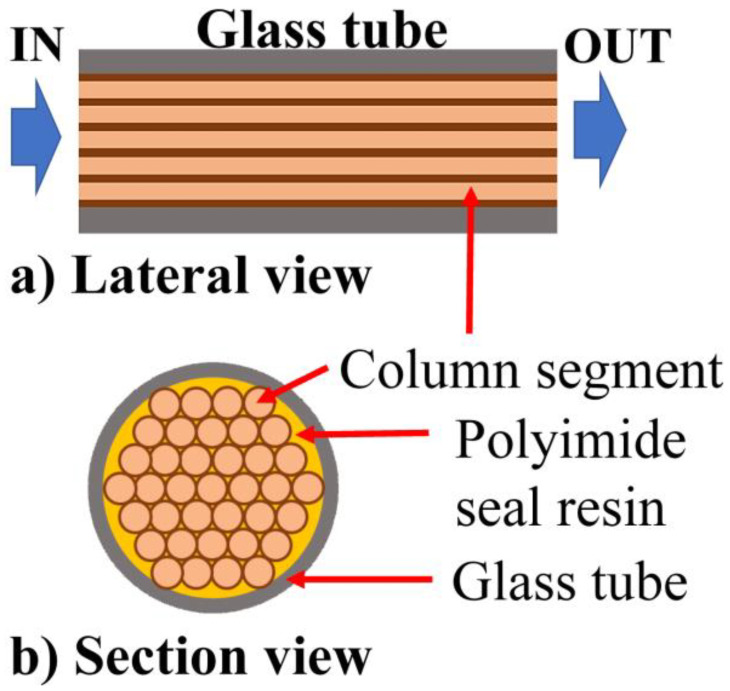
Schematic representation of lateral and frontal sectional views of the PCC.

The PCC consisted of a glass tube filled with a proper combination of 44 chromatographic column segments having an inner diameter of about 0.53 mm and a length of 100 mm. Each segment was cut from a ZB-624 column (by Zebron) suitable for VOCs and residual solvent analysis. This column has a stationary phase that is 3 µm thick, composed of 6% cyanopropylphenyl and 94% dimethylpolysiloxane. Its working temperature ranges between −20 to 260 °C (as described in column technical guide), and it works under an operative gas flow rate of about 2 mL/min. Using the proposed arrangement of column segments, the PCC could work at a maximum flow rate of 88 mL/min, calculated by multiplying the number of column sections (44) to a maximum single columns section flowrate (2 mL/min, as reported in the column technical guide). All presented tests were performed at lower flowrate (80 mL/min) for a conservative design rule. A flow meter and a proportional electric valve were used to control the flow stability. Teflon material was used both for the measure chamber (where the PID was allocated) and for all pneumatic components (e.g., tubing and connectors). Figure 3 shows a sketch of the PCC, where especially the lateral and the section views are represented. To avoid any kind of flow losses, polyimide sealing resin (by Sigma Aldrich) was used to seal all the interstices between the columns of the cartridge.

**Figure 4:**
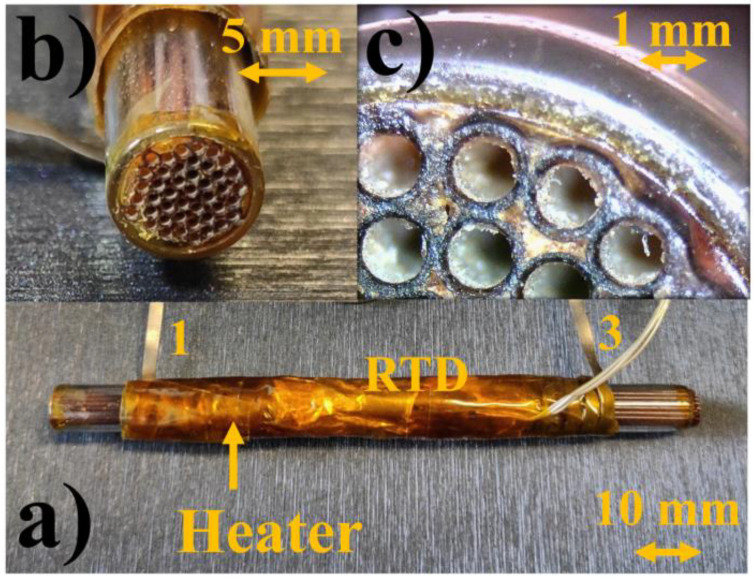
(**a**) Prototype of the Packed Columns Cartridge (PCC) with a rolled-up heater element and Resistance Temperature Detector (RTD). The final total PCC length was 80 mm. In particular, (1) and (3) wires were the heater extremities. (**b**) An extremity of the PCC and (**c**) a detail of the PCC segment column extremities.

In Figure 4 we show a photograph of the prototype of the PCC. Its fabrication process was performed in five main steps:

- Each chromatographic column segment, 100 mm long, was cut from the whole column by using a capillary GC column cutter.
- A cleaned glass tube, 80 mm long, having an inner and outer diameter of 5.5 mm and 7 mm, respectively, was filled with 44 column segments.
- Polyimide sealing resin (23,817 by Supelco) was used to fill all interstices between both the glass tube and the columns and between the columns themselves. Moreover, the resin was abundantly applied to the two extremities of the cartridge to facilitate the final cutting process.
- A diamond rotary saw was employed to cut the cartridge, in order to obtain a total length of 80 mm.
- A flexible heater was rolled up around the PCC and a Resistance Temperature Detector (RTD) model PT1000) was inserted to control the PCC temperature (see Figure 4a).

In order to reduce the error between the inner and outer tube working temperature, a set of calibration test were performed. The PCC was connected to the pneumatic system by using Teflon tube adapters suitable for high temperature application (e.g., denuders, traps, …). In order to test the PCC separation performances and the overall system functionality, we injected the desired volume of VOCs into the system inlet. The resulted data were elaborated online by LabView software that calculated the peak areas, starting from the time of injection.

### 2.2. Measurement System Testing

Benzene, toluene and p-xylene commercial laboratory standards (Benzene, 99.9+% HPLC grade; Toluene, ≥99.9% HPLC grade; p- Xylene, anhydrous, ≥99% all provided by SIGMA-ALDRICH) were used alone and to prepare the BTX mixture. The separation among the xylene isomers was not in the scope of the test, so only one of them was used. Acetone (for HPLC, ≥99.8%, by SIGMA-ALDRICH) and ethanol (for HPLC, ≥99.8%, by SIGMA-ALDRICH) were used to test possible interferences. Before all the experiments, the cartridge was heated, using the rolled-up heater, up to 240 °C to perform a cleaning procedure. During all the experiments, the cartridge temperature was maintained at 25 °C to have low power consumption, which fit with the aims of the proposed system. All the reported measurements were carried out in a climatic chamber, containing the whole system, with an average ambient temperature of about 25 °C to minimize the sample condensation phenomena.

A gas-tight syringe was used to inject a desired mass of the measured compound inside the system inlet. The injected sample was carried into the PCC by a flow (80 mL/min) generated by the pump. The desired mass (md), corresponding to a determinate volume (Vd) of saturated vapor, was withdrawn from the head space of the vial (containing the compounds) and kept at constant temperature (22 ± 0.1 °C) to avoid vapor pressure variations. The values of md were estimated utilizing the general gases equation taken into account: the compounds vapor pressure [53], vial temperature, ideal gas constant and Vd.

## 3. Results and Discussion

**Figure 5:**
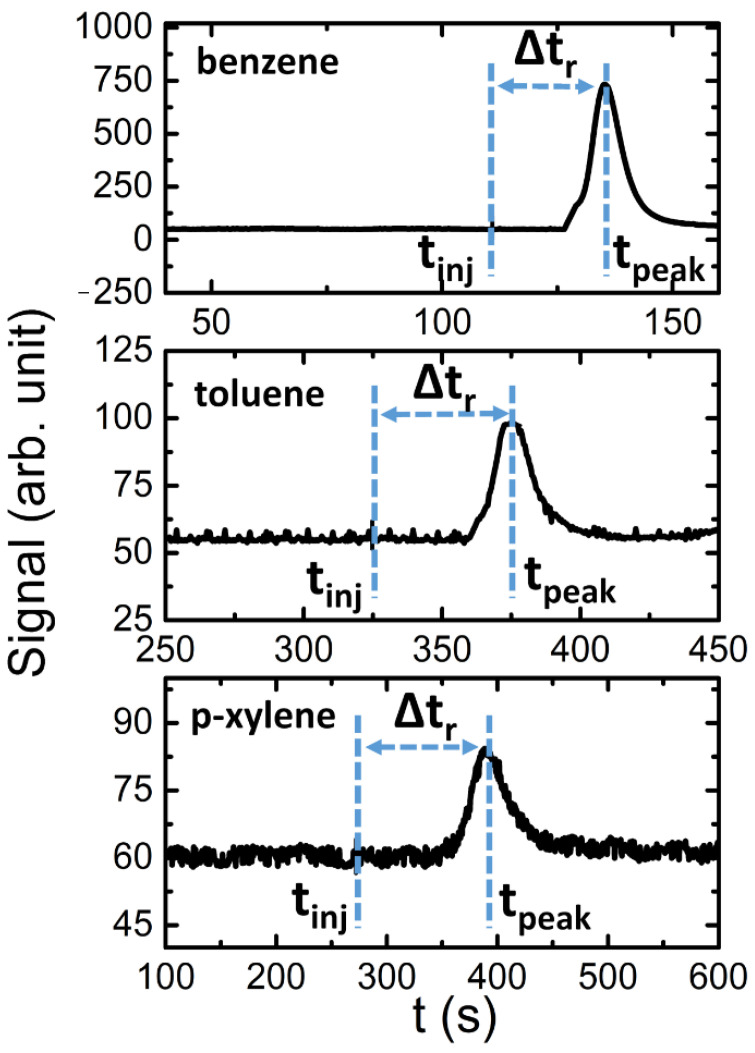
An example of BTX peaks, with 1.7 µg of benzene, 0.3 µg of toluene and 0.9 µg of p-xylene. In the chronograms, the instant time of injection (t_inj_), the evaluated instant of signal peak (t_peak_) and the relative retention time (Δtr) were drawn.

The results of the tests performed injecting the sample of benzene, toluene and p-xylene are shown first. Figure 5 reports an example of PID signals obtained after a sample injection of benzene (1.7 µg), toluene (0.3 µg) and p-xylene (0.9 µg), tested separately.

In order to calculate the retention time, Δt_r_ (which is defined as the difference between t_inj_ and t_peak_) for all the tested analytes, 20 repetitions at different masses for each compound have been performed. At each injection, the t_inj_ instant was recorded in the measurement file by a marker. From these experiments, we obtained a mean retention time value of 25 ± 0.5 s, 51 ± 1.2 s and 117 ± 4 s (see Figure 5) and a mean Full Width at Half Maximum (FWHM) of 8 s, 15 s and 40 s for the benzene, toluene and xylene, respectively. These results seem to confirm that the PCC works as a chromatographic device, performing an effective separation of the studied compounds.

**Figure 6:**
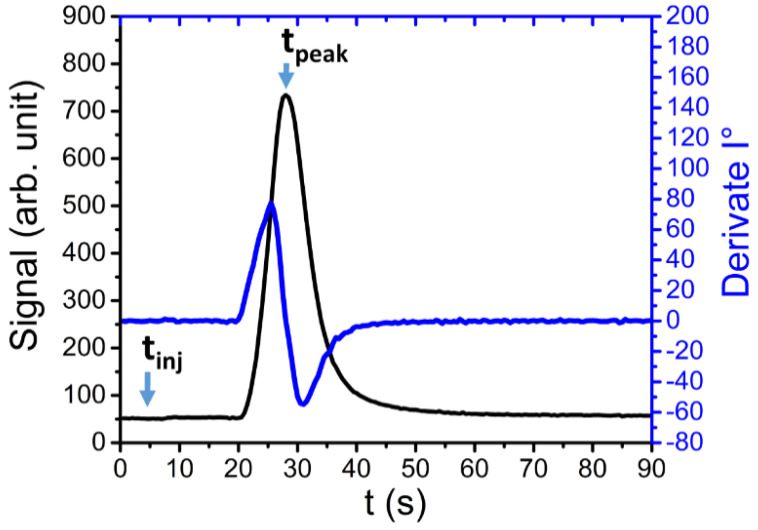
A detail of a benzene peak. In the Figure, t_inj_ and t_peak_ represent the injection and the peak instant, respectively. The peak Full Width at Half Maximum (FWHM) is about 8 s.

As highlighted in the following graph (Figure 6), after the injection the compound is retained by the PCC, and after a characteristic elapsed time (retention time), the substance arrives at the PID that detects the correlated peaks. A detailed chronograph of benzene peak and its first order derivate is also reported. The two important values *t_inj_* and *t_peak_*, respectively, refer to the injection and peak instant times. In this case, the peak FWHM is about 8 s.

In our case, from an initial analysis, the first order derivative highlights a non-perfect symmetry of all detected peaks (e.g., benzene, toluene and xylene). This result could be related to several parameters, such as the PCC length, a non-homogeneous heating of PCC and the performances spread due to the way in which the column sections work in parallel. The peak of the first order derivative is used to analyze several aspects of the separation process (or column performances), but this is not within the scope of the current work and could be the aim of a future study [54].

To study the PCC behavior at different flow rates, we have also performed the same experiments decreasing the PCC flow. In this case, the results showed that the PCC works perfectly and the retention times increased up to 100%. Especially in the case of benzene, by applying a flow rate of 8 sccm (1/10 of the original flow rate), the retention time was about 51 s. Although the results show that a lower flow rate enhances the PCC separation performances, the controlling system for low flow is more complicated and expensive. For these reasons, we set the flow rate at 80 sccm. In addition, a simple method to enhance the system performance in terms of separation could be to arrange several PCCs in series.

**Figure 7:**
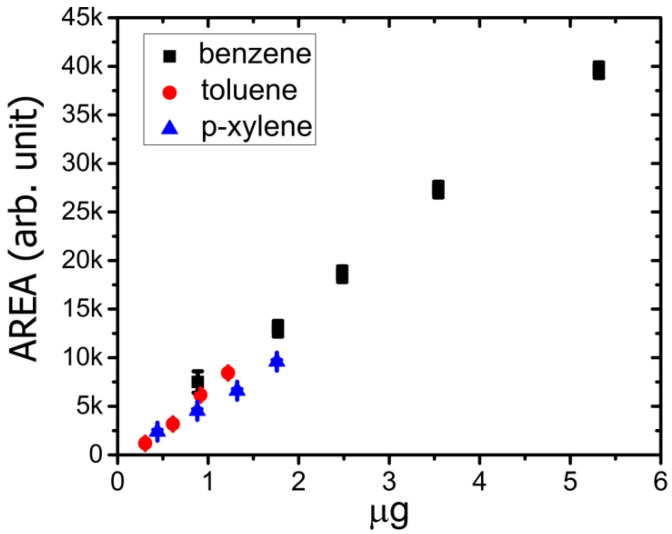
Response vs. injected mass of BTX. In particular, the peak areas versus the amount of compound in the injected sample (expressed in µg) are shown, including the error bar.

Figure 7 shows the relationship between the peak areas (evaluated by Origin Lab peak tools) versus the amount of compound in the injected sample (expressed in µg). The plotted data are the mean values of the experimental results measured for each concentration of BTX. The data were evaluated considering the PID response correction factor for each different analyte (ref TA02, https://www.ionscience.com).

From Figure 7, it is possible to see the quite linear behavior of the whole system. This result could be expected from both linear behaviors of the PID response and from the characteristics of the column used.

**Figure 8:**
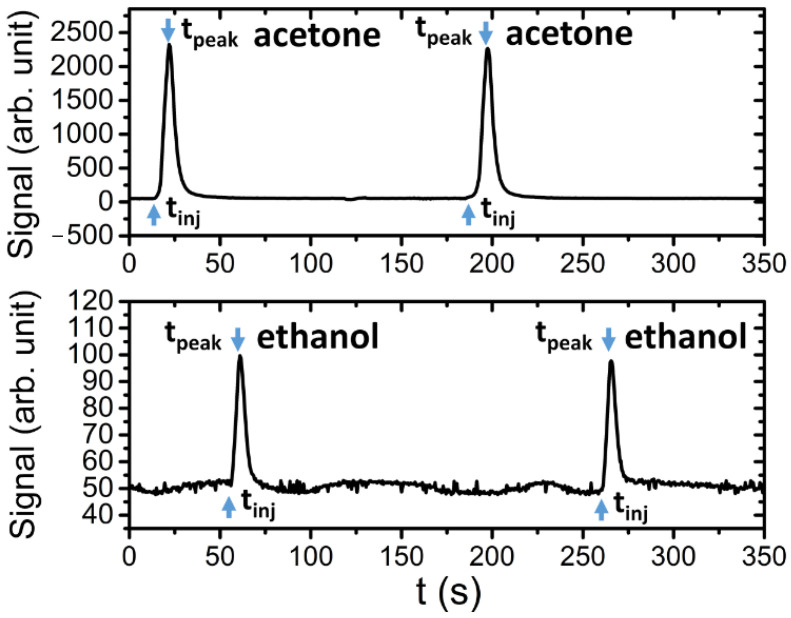
Example of peaks detected for ethanol and acetone.

When the developed systems have to be used for measurements in work environments (e.g., manufacturing industry, mechanics and shipyards) or in research, there could be various interferents, such as common solvents. For this reason, samples of acetone and ethanol have been injected in the system at fixed concentrations. Figure 8 reports an example of two successive peaks obtained injecting about 311 µg and 370 µg of acetone and ethanol, respectively.

**Figure 9:**
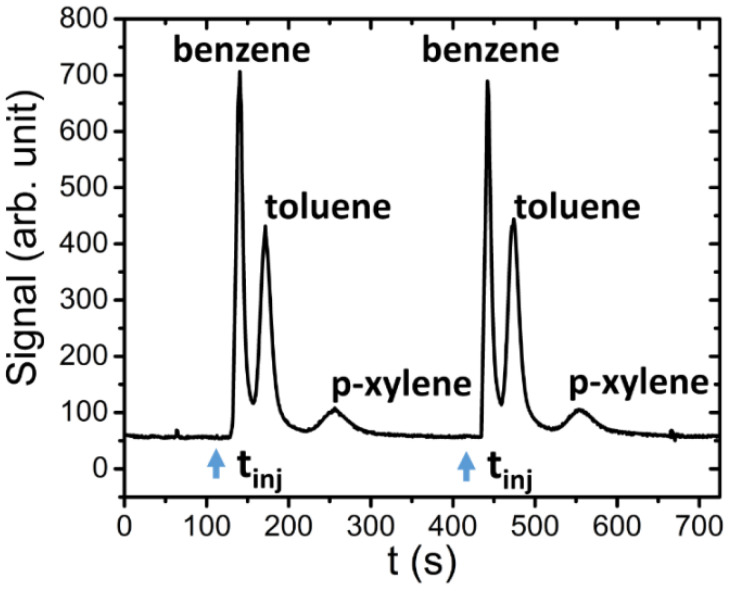
Example of two repetitions of a mixture injection (1.7 µg of benzene, 1.2 µg of toluene and 1.5 µg of p-xylene).

As is possible to see from the two plots in Figure 8, the retention times for these two compounds are very small (about 5 s). This behavior is due to the properties of the stationary phase used in the column sections. The BTX mixture was then injected into the system in order to verify its separation performances. The chronograph reported in Figure 9 presents two following injections of the BTX mixture (1.7 µg of Benzene, 1.2 µg of Toluene and 1.5 µg of p-xylene).

**Figure 10:**
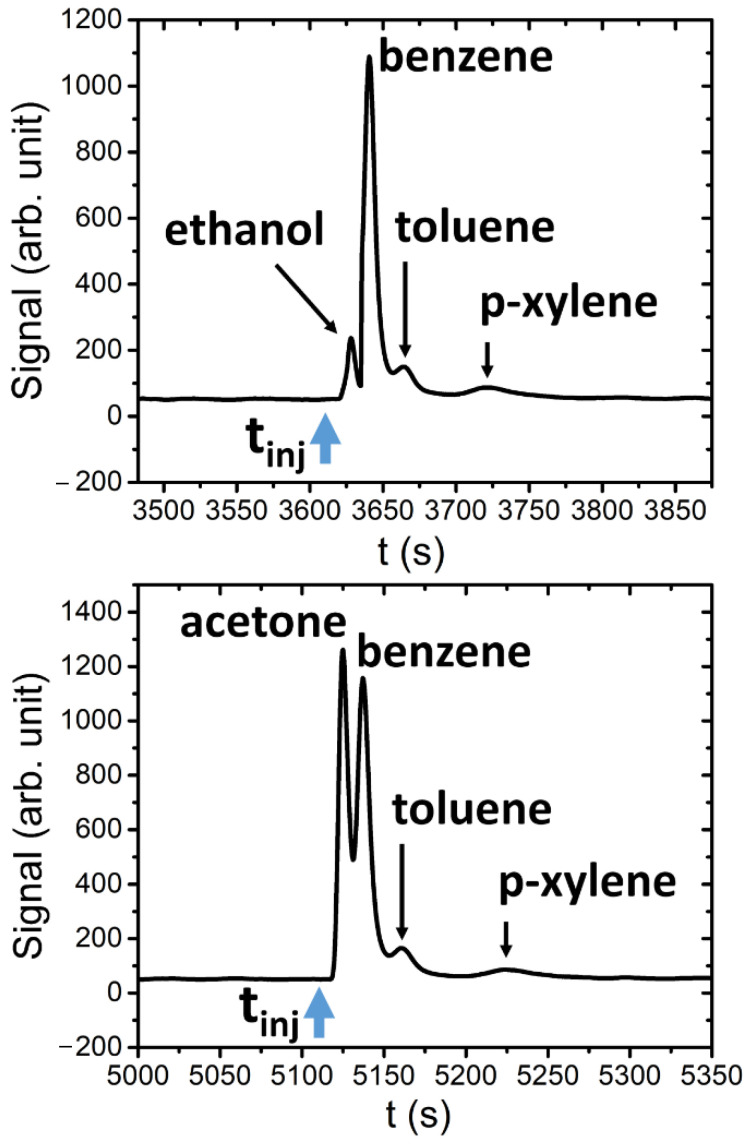
Example of two mixtures of ethanol, benzene, toluene and p-xylene (top graph) and acetone, benzene, toluene and p-xylene (bottom graph).

As is possible to observe, starting from the same t_inj_ (instant time of injection), the column separates the BTX components that are retained for different times (Δt_r_) before reaching the PID detector and being singularly revealed. Finally, in Figure 10 the results are reported that were obtained by injecting into the system two different mixtures: ethanol (765 µg), benzene (2.4 µg), toluene (0.4 µg) and p-xylene (0.9 µg), and acetone (160 µg), benzene (2.4 µg), toluene (0.4 µg) and p-xylene (0.9 µg).

The separation ability of developed system is highlighted in Figure 10. In fact, it is possible to distinguish the peaks, with a characteristic retention time (Δt_r_), for each mixture compound. From the experimental results, we can calculate the limit of detection (for BTX) by taking into account the signal to noise ratio (in a first approximation case = 3). The limit of detection value was about 0.03 µg, 0.1 µg and 0.5 µg for benzene, toluene and xylene, respectively. Finally, the linear behavior of the proposed system depends on column characteristics and PID performances. From data reported in Figure 7, the linearity (*R*-square = 0.991) of the whole setup chain was in the range of 0.3 µg to a few µg.

## 4. Conclusions

In the measurement and analysis of BTX, GC is a well-known technique, which provides a high accuracy, high selectivity and a low detection limit. Nevertheless, this technique also presents some drawbacks related to its high costs, size, weight and lack of portability. The availability of a reliable and low-cost portable GC for air monitoring could be of interest of smalland mediumsized enterprises for the monitoring of safe working conditions, and for air monitoring in developing countries. Hence, this work presents the development of a new BTX detection system, based on a mini chromatographic cartridge, a PID, an Arduino unit control and a membrane pump. This system is characterized by small dimensions (250 × 250 mm, not yet fully optimized), low weight (about 1 kg), easy portability, low-cost production (about 1000 €), simple usage and low energy consumption (about 2W), with a good accuracy and selectivity for the BTX analytes. The developed cartridge (80 mm in length) presents a good response towards the analytes, operating a separation of the injected BTX mixture, in the range of 0.3 to 5.3 µg. The separation performed by the cartridge showed a retention time of 25 ± 0.5 s, 51 ± 1.2 s and 117 ± 4 s for benzene, toluene and p-xylene, respectively. The limit of the detection value (for BTX, by taking into account the signal to noise ratio = 3) was about 0.03 µg, 0.1 µg and 0.5 µg for benzene, toluene and xylene, respectively. Finally, the linearity (*R*-square = 0.991) of the whole setup chain was in the range of 0.3 µg to a few µg.

This result allowed us to clearly distinguish the different analytes present in the injected mixture and to quantify their concentrations.

The performances of the system give us the opportunity to continue to work on the system, implementing and investigating some aspects of the separation process, such as the examination of the first order derivative peaks, trying to enhance the cartridge and all the measurement system performances. Further investigations could be focused on the monitoring of different mixtures, typical of selected workplaces, such as styrene in the fiberglass industry, of disinfectants and anesthetics in healthcare environments.

## Data Availability

The data presented in this study are available on request from the corresponding author.

## References

[1] WHO (2010). Selected Pollutants (WHO Guidelines for Indoor Air Quality).

[2] Kim D., Chen Z., Zhou L.-F., Huang S. (2018). Air pollutants and early origins of respiratory diseases. Chronic Dis. Transl. Med..

[3] Szczurek A., Maziejuk M., Maciejewska M., Pietrucha T., Sikora T. (2017). BTX compounds recognition in humid air using differential ion mobility spectrometry combined with a classifier. Sens. Actuators B Chem..

[4] Kim K.-H., Pandey S.K., Pal R. (2009). Analytical bias among different gas chromatographic approaches using standard BTX gases and exhaust samples. J. Sep. Sci..

[5] Schnatter A.R., Kerzic P.J., Zhou Y., Chen M., Nicolich M.J., Lavelle K., Armstrong T.W., Bird M., Lin L., Fu H. (2010). Peripheral blood effects in benzene-exposed workers. Chem. Interact..

[6] Masih A., Lall A., Taneja A., Singhvi R. (2018). Exposure levels and health risk assessment of ambient BTX at urban and rural environments of a terai region of northern India. Environ. Pollut..

[7] Wilbur S.B., Keith S., Faroon O., Wohlers D. (2007). Toxicological Profile for Benzene.

[8] Gallego E., Roca F., Guardino X., Rosell M. (2008). Indoor and outdoor BTX levels in Barcelona City metropolitan area and Catalan rural areas. J. Environ. Sci..

[9] Mirzaei A., Leonardi S.G., Neri G. (2016). Detection of hazardous volatile organic compounds (VOCs) by metal oxide nanostruc-tures-based gas sensors: A review. Ceram. Int..

[10] Thammakhet C., Muneesawang V., Thavarungkul P., Kanatharana P. (2006). Cost effective passive sampling device for volatile organic compounds monitoring. Atmos. Environ..

[11] Thammakhet C., Villeneuve T., Munisawang V., Thavarungkul P., Kanatharana P. (2004). Monitoring of BTX by passive sampling in Hat Yai. J. Sci. Technol..

[12] Du Z., Mo J., Zhang Y., Li X., Xu Q. (2013). Evaluation of a new passive sampler using hydrophobic zeolites as adsorbents for exposure measurement of indoor BTX. Anal. Methods.

[13] Szulczyński B., Gębicki J. (2017). Currently Commercially Available Chemical Sensors Employed for Detection of Volatile Organic Compounds in Outdoor and Indoor Air. Environments.

[14] Spinelle L., Gerboles M., Kok G., Persijn S., Sauerwald T. (2017). Review of Portable and Low-Cost Sensors for the Ambient Air Monitoring of Benzene and Other Volatile Organic Compounds. Sensors.

[15] Su Y., Yang T., Zhao X., Cai Z., Chen G., Yao M., Chen K., Bick M., Wang J., Li S. (2020). A wireless energy transmission enabled wearable active acetone biosensor for non-invasive prediabetes diagnosis. Nano Energy.

[16] Persaud K.C. (2005). Polymers for chemical sensing. Mater. Today.

[17] Fratoddi I., Altamura P., Bearzotti A., Furlani A., Russo M. (2004). Electrical and morphological characterization of poly(monosubstituted)acetylene based membranes: Application as humidity and organic vapors sensors. Thin Solid Films.

[18] Li W., Wu X., Han N., Chen J., Qian X., Deng Y., Tang W., Chen Y. (2016). MOF-derived hierarchical hollow ZnO nanocages with enhanced low-concentration VOCs gas-sensing performance. Sens. Actuators B Chem..

[19] Huang H., Xu P., Zheng D., Chen C., Li X. (2014). Sulfuration–desulfuration reaction sensing effect of intrinsic ZnO nanowires for high-performance H2S detection. J. Mater. Chem. A.

[20] Manzo C., Mei A., Zampetti E., Bassani C., Paciucci L., Manetti P. (2017). Top-down approach from satellite to terrestrial rover application for environmental monitoring of landfills. Sci. Total Environ..

[21] Mirzaei A., Kim J.H., Kim H.W., Kim S.S. (2018). Resistive-based gas sensors for detection of benzene, toluene and xylene (BTX) gases: A review. J. Mater. Chem. C.

[22] Bearzotti A., Macagnano A., Papa P., Venditti I., Zampetti E. (2017). A study of a QCM sensor based on pentacene for the detection of BTX vapors in air. Sens. Actuators B Chem..

[23] Viespe C., Grigoriu C. (2010). Surface acoustic wave sensors with carbon nanotubes and SiO2/Si nanoparticles based nanocomposites for VOC detection. Sens. Actuators B Chem..

[24] Penza M., Antolini F., Antisari M. (2004). Carbon nanotubes as SAW chemical sensors materials. Sens. Actuators B Chem..

[25] De Cesare F., Di Mattia E., Pantalei S., Zampetti E., Vinciguerra V., Canganella F., Macagnano A. (2011). Use of electronic nose technology to measure soil microbial activity through biogenic volatile organic compounds and gases release. Soil Biol. Biochem..

[26] Hamdi K., Hébrant M., Martin P., Galland B., Etienne M. (2016). Mesoporous silica nanoparticle film as sorbent for in situ and real-time monitoring of volatile BTX (benzene, toluene and xylenes). Sens. Actuators B Chem..

[27] Hue J., Dupoy M., Bordy T., Rousier R., Vignoud S., Schaerer B., Tran-Thi T.-H., Rivron C., Mugherli L., Karpe P. (2013). Benzene and xylene detection by absorbance in the range of 10–100ppb application: Quality of indoor air. Sens. Actuators B Chem..

[28] Camou S., Shimizu A., Horiuchi T., Haga T. (2008). ppb-Level detection of benzene diluted in water with portable device based on bubbling extraction and UV spectroscopy. Sens. Actuators B Chem..

[29] Azzouz A., Vikrant K., Kim K.-H., Ballesteros E., Rhadfi T., Malik A.K. (2019). Advances in colorimetric and optical sensing for gaseous volatile organic compounds. TrAC Trends Anal. Chem..

[30] Khan S., Le Calvé S., Newport D. (2020). A review of optical interferometry techniques for VOC detection. Sens. Actuators A Phys..

[31] Peng F.-M., Xie P., Li H., Zhang Y.-H., Wang J.-D., Liu W.-Q. (2008). Effect of Atmospheric Interfering Absorption on Measurement of BTX by DOAS. Chin. J. Chem. Phys..

[32] Oser H., Coggiola M.J., Young S.E., Crosley D.R., Hafer V., Grist G. (2007). Membrane introduction/laser photoionization time-of-flight mass spectrometry. Chemosphere.

[33] Bottein M.-Y.D., Fuquay J.M., Munday R., Selwood A.I., Van Ginkel R., Miles C., Loader J.I., Wilkins A.L., Ramsdell J.S. (2010). Bioassay methods for detection of N-palmitoylbrevetoxin-B2 (BTX-B4). Toxicon.

[34] Grob R.L., Barry E.F. (2004). Modern Practice of Gas Chromatography.

[35] Snyder L.R., Kirkland J.J., Dolan J.W. (2009). Introduction to Modern Liquid Chromatography.

[36] Poole C. (2012). Gas Chromatography.

[37] Bahrami A., Mahjub H., Sadeghian M., Golbabaei F. (2011). Determination of Benzene, Toluene and Xylene (BTX) Concentrations in Air Using HPLC Developed Method Compared to Gas Chromatography. Int. J. Occup. Hyg..

[38] Grametbauer P., Kartusek S., Hausner O. (1988). Diagnosis of aerobic gram-negative bacteria by the detection of volatile metabolites using gas chromatography. Mikrobiol. Imunol..

[39] Phillips M. (1997). Method for the Collection and Assay of Volatile Organic Compounds in Breath. Anal. Biochem..

[40] Saka M. (2010). Application of gas chromatograph with selected detector for pesticide residue analysis in food. J. Pestic. Sci..

[41] Wang Z. (2003). Fate and Identification of Spilled Oils and Petroleum Products in the Environment by GC-MS and GC-FID. Energy Sources.

[42] Wu B.-Z., Feng T.-Z., Sree U., Chiu K.-H., Lo J.-G. (2006). Sampling and analysis of volatile organics emitted from wastewater treatment plant and drain system of an industrial science park. Anal. Chim. Acta.

[43] Dziuban J., Mroz J., Szczygielska M., Małachowski M., Gorecka-Drzazga A., Walczak R., Buła W., Zalewski D., Nieradko Ł., Łysko J. (2004). Portable gas chromatograph with integrated components. Sens. Actuators A Phys..

[44] Radadia A.D., Morgan R.D., Masel R., Shannon M.A. (2009). Partially Buried Microcolumns for Micro Gas Analyzers. Anal. Chem..

[45] Kaanta B.C., Chen H., Zhang X. (2010). A monolithically fabricated gas chromatography separation column with an integrated high sensitivity thermal conductivity detector. J. Micromech. Microeng..

[46] Agah M., Wise K.D. (2007). Low-Mass PECVD Oxynitride Gas Chromatographic Columns. J. Microelectromech. Syst..

[47] Zampolli S., Elmi I., Cardinali G.C., Masini L., Bonafè F., Zardi F. (2020). Compact-GC platform: A flexible system integration strategy for a completely microsystems-based gas-chromatograph. Sens. Actuators B Chem..

[48] Kalsoom U., Nesterenko P., Paull B. (2018). Current and future impact of 3D printing on the separation sciences. TrAC Trends Anal. Chem..

[49] Contreras J.A., Murray J.A., Tolley S.E., Oliphant J.L., Tolley H.D., Lammert S.A., Lee E.D., Later D.W., Lee M.L. (2008). Hand-Portable Gas Chromatograph-Toroidal Ion Trap Mass Spectrometer (GC-TMS) for Detection of Hazardous Compounds. J. Am. Soc. Mass Spectrom..

[50] Mini GC Plus, by Vernier. https://www.vernier.com.

[51] Torion T-9 Portable GC/MS, by PerkinElmer. www.perkinelmer.com.

[52] Mini GC, by Forston Labs. https://www.analyticalwest.com/mini-gc.html.

[53] NIST Chemistry WebBook. https://www.nist.gov/.

[54] Wahab M.F., Patel D.C., Armstrong D.W. (2017). Total peak shape analysis: Detection and quantitation of concurrent fronting, tailing, and their effect on asymmetry measurements. J. Chromatogr. A.

